# The time is ripe for the renaissance of autism treatments: evidence from clinical practitioners

**DOI:** 10.3389/fnint.2023.1229110

**Published:** 2023-08-04

**Authors:** Elizabeth B. Torres, Goldie Twerski, Hannah Varkey, Richa Rai, Mona Elsayed, Miriam Tirtza Katz, Jillian Tarlowe

**Affiliations:** ^1^Sensory Motor Integration Laboratory, Department of Psychology, Rutgers the State University of New Jersey, Piscataway, NJ, United States; ^2^Rutgers Center for Cognitive Science, Rutgers the State University of New Jersey, Piscataway, NJ, United States; ^3^Department of Computer Science, Rutgers Center for Biomedicine Imaging and Modeling, Rutgers the State University of New Jersey, Piscataway, NJ, United States; ^4^The Monarch Center, Lakewood, NJ, United States; ^5^MTK Therapy, Yahalom NJ, Family Advocacy and Support, Agudas Yisroel of America, Lakewood, NJ, United States

**Keywords:** autism, applied behavioral analysis, DIR Floortime, developmental model, neurodevelopment, wearable biosensors

## Abstract

**Introduction:**

Recent changes in diagnostics criteria have contributed to the broadening of the autism spectrum disorders and left clinicians ill-equipped to treat the highly heterogeneous spectrum that now includes toddlers and children with sensory and motor issues.

**Methods:**

To uncover the clinicians’ critical needs in the autism space, we conducted surveys designed collaboratively with the clinicians themselves. Board Certified Behavioral Analysts (BCBAs) and developmental model (DM) clinicians obtained permission from their accrediting boards and designed surveys to assess needs and preferences in their corresponding fields.

**Results:**

92.6% of BCBAs are open to diversified treatment combining aspects of multiple disciplines; 82.7% of DMs also favor this diversification with 21.8% valuing BCBA-input and 40.6% neurologists-input; 85.9% of BCBAs and 85.3% of DMs advocate the use of wearables to objectively track nuanced behaviors in social exchange; 76.9% of BCBAs and 57.0% DMs feel they would benefit from augmenting their knowledge about the nervous systems of Autism (neuroscience research) to enhance treatment and planning programs; 50.0% of BCBAs feel they can benefit for more training to teach parents.

**Discussion:**

Two complementary philosophies are converging to a more collaborative, integrative approach favoring scalable digital technologies and neuroscience. Autism practitioners seem ready to embrace the Digital-Neuroscience Revolutions under a new cooperative model.

## 1. Introduction

**FIGURE 1:**
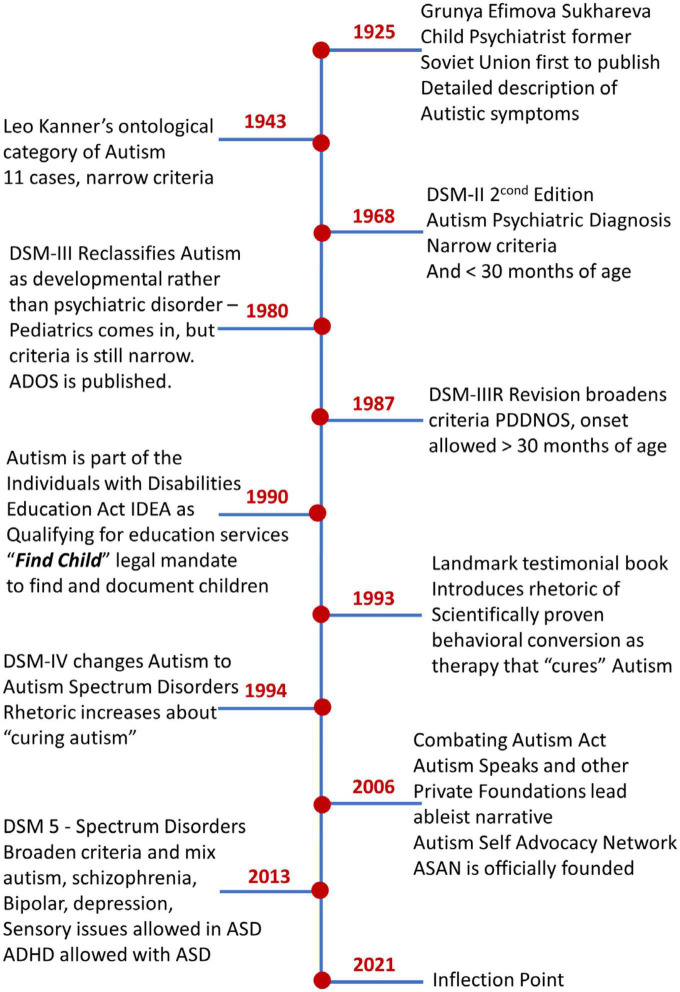
Historical context of ASD evolution highlighting shifts in diagnostics criteria and social landmarks influencing the epidemiology and the diagnosis-to-behavioral-treatment pipeline. While parents complained that the system gaslights them with information they cannot verify, mandates across the US continue to favor one type of behavioral therapy over others. Between the exponential rates of prevalence and the societal pressures, autism has arguably reached an inflection point tending to a more precision-, personalized medicine-based approach aimed at stratifying the spectrum and managing disorders by repurposing technology from other more scientifically and technologically advanced fields (Hawgood et al., 2015; Torres, 2020, 2021) that address nervous systems’ support and accommodations for daily life.

The autism diagnostics criteria have shifted over the years allowing a broader range of professionals to diagnose the condition, while also shifting the age of diagnosis to earlier developmental stages of infancy. From merely 11 cases recognized in 1943 by Dr. Leo Kanner’s narrowly defined, ontological category of Autism, to the recent shift from the Diagnostics Statistical Manual (American Psychiatric Association [APA], 2013), moving the DSM IV to the DSM 5, Figure 1, we have seen a dramatic change in the epidemiology of autism spectrum disorders. Part of the change, for instance, includes attention deficit hyperactivity disorder (ADHD) in the DSM-5, a condition that was not allowed in the DSM-IV criteria, to coexist with the ASD diagnosis. Likewise, the inclusion in the DSM-5 of sensory issues in the criteria for ASD, opens the condition to a broad segment of the population of children who suffer today from sensory over- or under-sensitivities e.g., linked to food and other environmentally triggered allergens (Xu et al., 2018). Adding to these shifts in DSM-based criteria for ASD, the Autism Diagnostics Observation Schedule [now in its second edition, ADOS-2 (Lord et al., 1989, 2000)] have produced new modules for toddlers (Esler et al., 2015) and adults (Hus and Lord, 2014), thus refining and extending the detection of ASD across the human lifespan, albeit with recognized statistical biases bound to inflate false positives in males (Torres et al., 2017, 2020b), while missing females (Mandy et al., 2012; Loomes et al., 2017; Lundstrom et al., 2019; D’Mello et al., 2022).

Surveillance in the US, following the guidelines of the Centers for Disease Control and Prevention, and by the World Health Organization, report an exponential rise in ASD prevalence (Christensen et al., 2019), with pronounced disparities, across low-income families and underrepresented sectors of the population (Maenner et al., 2020; Zhou et al., 2023), raising concerns about ASD detection and access to treatment. While arguably the shifts in criteria and the improvements in early detection have generally led to the exponential prevalence rates, the professional workforce, and the availability (in the US) of medical insurance coverage for some autism interventions have proven insufficient to provide proper support and accommodations to the highly heterogeneous population of ASD.

The availability of insurance coverage for ASD therapies was already constrained to exclusively cover behavioral intervention in the USA, i.e., applied behavioral analysis (ABA), leaving other interventions at a disadvantage, as they are not affordable by a large majority of the population.^1^ With a minimum average of 60K per child/household in out-of-pocket expenses for other therapies and for additional sessions of ABA,^2^ most families cannot afford the diversification of therapies that they need to help toddlers, children with a broad spectrum of sensory and motor issues, and with coexisting ADHD-ASD conditions (Rommelse et al., 2010; Leitner, 2014; Polderman et al., 2014; Zablotsky et al., 2020; Zhou et al., 2023). The latter includes individuals with conditions like obsessive compulsive disorder (OCD) (van der Plas et al., 2016), a genetic predisposition to Post-Traumatic Stress Syndrome (PTSD) (Torres, 2021) and other motor-related conditions involving cerebellar dysfunction (Mosconi et al., 2015) and dystonia (Xiao et al., 2020), motor tics (Bell et al., 2019), vestibular disorders (Bermperidis et al., 2021), ataxias (Pedapati et al., 2019; Cabal-Herrera et al., 2020a,b; Torres, 2020; Zafarullah et al., 2020), excess involuntary micro-movements (Torres and Denisova, 2016; Torres et al., 2017; Caballero et al., 2018), among others. These differences in neurodevelopment are bound to interfere with activities of daily life. Furthermore, they impede social interactions and communications, and explain many causes behind repetitive motions, all part of the official, current criteria for ASD (American Psychiatric Association [APA], 2013). Adding to these co-existing conditions are rare genetic disorders and disorders of known etiology, like Down Syndrome (Rasmussen et al., 2001; Bradbury et al., 2021), SHANK3 deletion syndrome (Phelan et al., 1993; Torres et al., 2016a; Schaffler et al., 2019), Fragile X (Kaufmann et al., 2017), various forms of Cerebral Palsy (Pahlman et al., 2020, 2021; Gecz and Berry, 2021), just to name a few.

Two types of therapies available today have complementary philosophies and very different curricula for accreditation. At one end, we have the developmental model (DM)-based therapies that emphasize relationships, affect and playful exploratory learning allowing the young nervous system to self-discover purposeful behaviors, while using seemingly purposeless interactive motions. They encourage the self-emergence of rapport, turn-taking, attentional modulation, and emotional regulation between the infant and the caregiver or therapist. These aspects of social interaction are critical during early neurodevelopment of a nascent nervous system. They however, pose many challenges to developmental clinicians when trying to develop reliable and systematic consensus representing criteria for functional milestones. At the other end, classical ABA relies more on structured environments and prompts that enable the child to follow instructions and complete goal-directed actions, but inevitably sideline more ambiguous, nuanced aspects of naturalistic behaviors and social interactions that transpire largely beneath awareness and as such, escape the naked eye of the therapist guiding the social interaction with the child. Most importantly, the reward-based conditioning of discretized actions impedes the self-emergence of spontaneous autonomy -a key ingredient in socio-motor agency (Torres et al., 2013b). Although recent ABA models have focused more heavily on NET (Naturalistic Environmental Teaching) models, the emphasis on data driven goals rather than process-based interactions leads to a model driven by the outcome or resulting discrete behavior, rather than the *process* of spontaneous self-discovery, learning and developing without being conditioned to do so.

Earlier practices of ABA and their scarce scientific evidence from human development have raised ethical concerns (Gernsbacher, 2006; Wilkenfeld and McCarthy, 2020; Bottema-Beutel and Crowley, 2021; Leaf et al., 2022). Most importantly they have been associated with trauma (Stack and Lucyshyn, 2019; Leaf et al., 2022), a recognition that have prompted changes in modern versions of cognitive behavioral therapy geared to treat the anxiety linked with such excess control over the minor during the treatments (Ung et al., 2015). Infringement of human agency from an early age, regardless of the therapeutic intervention that does it, ought to be a major concern for all involved in deciding and consenting on behalf of the minor. However, clinical interventions in the US are guided, approved, and supervised by the accrediting bodies of the intervention itself, rather than by multidisciplinary committees with no skin in the game, as those in Institutional Review Boards that supervise research practices. Indeed, none of the trauma-inducing scenarios described by self-advocates and parents would have taken place at a research laboratory where IRB-approved protocols instruct immediately discontinuing any task that shows discomfort to the minor. A question then is why are accrediting clinical organizations not following basic IRB research stipulations compliant with the Helsinki Act? Indeed, at present in the US, clinical practices do not have to abide by the same guidelines that researchers are bound to follow.

**FIGURE 2:**
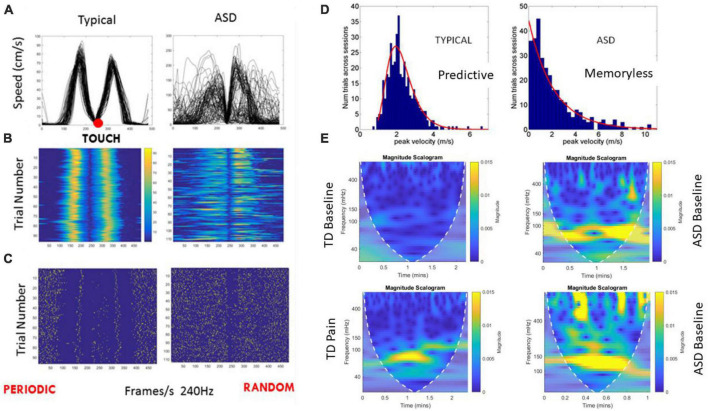
Dysregulation of motor reafference sensory feedback and autonomic activities in ASD. **(A)** Reaching hand speed vs. time profiles in route to touching a target and spontaneously, without instruction, moving the hand back toward rest, over 100 repetitions. Typical profile consists of a raising acceleration phase to the single peak, followed by a deceleration phase, slowing down to the target, and briefly pausing before returning the hand back to rest. The trial-to-trial variability gives rise to consistent reafferent motor feedback, which enables the child to predict the consequences of one’s self-generated action, thus building a sense of action ownership and enabling predictive motor control. Approximately symmetric, bell-shaped speed profiles are a signature of smooth, voluntary motions (Viviani and Flash, 1995). **(B)** Heat maps of the peaks show the highly predictable consistency of the typical patterns, from trial to trial, contrasting with the dysregulated, highly uncertain ASD pointing patterns. **(C)** Spike trains representation of the micro-movements derived from the peak variability (Torres et al., 2013a; Wu et al., 2018) also shows the periodic, predictable typical case *vs.* the random noise in the ASD case. **(D)** These trial-to-trial speed peaks are gathered in a frequency histogram that shows the memoryless, random nature of the motor reafference (feedback) in ASD contrasting with the predictable voluntary motor code. This result consistently found in ASD from 3 to 60 years of age, explains the disconnect between the mental intent to move, and the physical lack of volition elegantly explained by autistic individuals through self-reports. **(E)** Autonomous system dysregulation in ASD can be measured with a scalogram plot contrasting the frequency landscape of the inter beat interval (IBI) timing variability for a typically developing (TD) case, unambiguously differentiating between resting state and pain state (abnormally high low frequency band signature of flight-or-fight state, characterizing stress, and high anxiety levels). Consistently, ASD children at baseline, resting state, boast the pain, stress, anxiety signature of the heart’s IBI timing variability.

Both therapies are important, yet complementary. Both therapies rely on subjective (manualized) evaluations of behavior. Neither currently rely on objective physiologically-based biometrics characterizing nervous systems functions [e.g., (Torres, 2018)]. Furthermore, research suggests that they ought to be administered according to the human ontological orderly maturation of the nervous system, rather than imposed *a priori* on a young child, without first understanding the internal states of the nascent nervous system -particularly that of a human infant whose nervous system has taken a different path altogether (Torres et al., 2016b,^2023^). For example, Figure 2 shows commonly found dysregulated activities from the autonomic and voluntary systems of representative individuals with ASD *vs.* age-matched neurotypical children. Overall, these data demonstrate easily-accessible physiological responses that have been shown to objectively differentiate between autistic and neurotypical children.

Based on survey responses, clinicians from both fields expressed a need for a different type of training and support, to manage the new wave of diagnosed ASD. However, no consensus has been reached yet about their technological and scientific needs, by the respective leading organizations that confer accreditation to these health professionals operating under behavioral principles and manualized measuring techniques developed in the 1950s–1970s period. While each group may think that the other’s approach is at odds with the underlying principles of their own philosophy, many now recognize the importance of the other’s methods. Here we show this surprising outcome from surveys that these two camps of clinicians designed, supported by the New Jersey Autism Center of Excellence (tenured from 2018 to 2023 under the support of the New Jersey Governor’s Council for Autism and the New Jersey Department of Health) and under the leadership of the first author.

To avoid confirmation bias in each of the presented surveys, their designs were led by those from the corresponding fields, rather than from the authors themselves. The context in which these surveys took place, and the motivation behind them, came from state-wide polls in New Jersey, USA, focus groups on New Jersey, USA, and 118 nationwide interviews from the National Science Foundation Innovation Corps, to uncover market fit for technology developed by the first author’s lab. The NSF interviews serendipitously led from college athletics to the ASD ecosystem.^3^ The interviews revealed the lack of insurance coverage for ASD therapies (other than ABA), in addition to revealing their lack of curricula on the nervous systems’ development along with the prevalence of subjective metrics that insurance companies had started to suspect were insufficient to show therapy effectiveness. In turn, the latter led to the discovery of an alternative set of developmental therapies for ASD that despite lack of insurance coverage, were being sought out by parents with financial means who could afford them. In addition, there were reports by BCBA’s in private practices utilizing a range of developmental knowledge and techniques together with their behavioral methodologies and practice.

The above NSF I-Corps and NJACE focus group interviews led to a state-wide poll in New Jersey, USA, revealing that 80% New jerseyans knew an autistic individual, believed that ASD was a behavioral problem and had heard that ASD is a disorder of the brain.^4^ Motivated by these outcomes, we launched the two surveys by BCBAs and DM clinicians. Both therapists interact with different age groups in autism, representative of issues across the human lifespan. We report the outcomes of both surveys and discuss the possible consequences for ASD research and therapies.

## 2. Materials and methods

This survey study was approved by the Rutgers University Institutional Review Board, following the tenets of the Helsinki Act.

### 2.1. BCBA demographics

The BCBA survey was placed online through SurveyMonkey, with a link sent to BCBAs through the BACB mass email service. It was taken by 70 BCBAs from the state of New Jersey and by 102 additional participants from the rest of the continental USA, for a total of 172 BCBAs.

### 2.2. DM demographics

The DM survey was placed online through SurveyMonkey with a link sent via email to Profectum and ICDL certificate databases. It was taken by 170 participants, 7 from New Jersey and the rest from the continental USA and Canada. In this group, 51.8% remained anonymous and 48.82% shared their names. The survey was approved by the International Council on Development and Learning (ICDL), offering DIR Floortime^®^ therapy. Feedback from Dr. Serena Weider, co-inventor of the model with the late Dr. Stanley Greenspan, was also obtained by the designers of the survey.

There was a rather varied composition of this group, 32.25% from OT/PT (the highest grouping since OT and PT overlapped and were combined), 25.29% Speech Language Pathologists, 18.24% education or special education, 16.47% mental health providers, and 7.65% unspecified. Of these respondents, 76 reported master’s level training, 22 reported bachelor’s degree, 16 reported PhD, 56 did not report their education level. Training level by ICDL standards was 51.8% basic level, 25.29% advanced and 23.53% expert level. The age of the population that they receive was broken down as 68.82% infants and toddlers; 87.65% pre-school students; 78.24% school age students and 16.47% work with adults. Many practitioners are working across more than one setting, so they operate across multiple zip codes across the USA and Canada. Among them, 61.8% work in clinics, 41.8% work in homes, 41.76% work in schools and 14.72% work in other, unspecified locations.

### 2.3. Analyze text data using topic models

We used a Latent Dirichlet Allocation (LDA) model to discover underlying topics in a collection of documents and to infer the word probabilities in topics. The answers to each question were tabulated in coma-separated-files and the data tokenized, normalized, and prepared for text analyses by eliminating punctuations, using the sample code below in Matlab R2020a (e.g., for answers to question 16 for ABA US folks). Upon generating topic word clouds unveiling themes across topics, probability graphs were also obtained. To decide on a suitable number of topics, we obtained goodness-of-fit of the LDA model fit using varying numbers of the emerging topics. Then we evaluated the goodness-of-fit of the LDA model by obtaining the measure of perplexity of a held-out set of documents (in a training-validation paradigm). The perplexity indicates how well the model describes a set of documents, with lower perplexity suggesting a better fit.

**Table:** 

data16 = readtable(“Q16 ABA US.csv,” “TextType,” “string”);
head (data16)
textData16 = data16.RESPONSES;
documents16 = preprocessText(textData16);
bag16 = bagOfWords(documents16)
bag16 = removeInfrequentWords(bag16,2);
bag16 = removeEmptyDocuments(bag16);
numTopics = 6;
mdl = fitlda(bag16,numTopics, “Verbose,”0);
figure;
for topicIdx = 1:numTopics
subplot(2,3,topicIdx)
wordcloud(mdl,topicIdx);
title(“Topic ” + topicIdx)
end
print -dtiff Topics_Q16_ABA_US;
% Probability bars
newDocument16 = tokenizedDocument (documents16);
topicMixture16 = transform (mdl,newDocument16);
figure
bar(topic Mixture16)
xlabel (“Topic Index”)
ylabel (“Probability”)
title(“Q16 ABA US-Document Topic Probabilities”)
print -dtiff Probability_Q16_ABA_US;

## 3. Results

The responses are presented first from the BCBA group, followed by those of the DM group, split into the Profectum Foundation and the International Council on Development and Learning (ICDL) fellows.

### 3.1. BCBAs responses

Q1 Do you consent to participate in this survey? 68/70 said Yes and 2 skipped it.

Q2 Zip code: 63 answered and 7 skipped it zip code distribution in Supplementary Appendix A.

Q3 Level of ABA Certification (64 answered and 4 skipped it): 1.56% were Registered Behavioral Technician (RBT), the lowest training category of the BCBA tier system, yet the most abundant in the school’s system of the US. These are required only a high school diploma; 28.13% were Board Certified assistant Behavioral Analyst (BCaBA) who need to hold a bachelor’s degree; 67.19% were BCBA with a master’s degree and 3.13% were BCBA-D with a PhD level.

Q4 Age of treatment population (65 answered and 5 skipped it). The age of treated population was highest at school age (90.77%); followed by pre-school age (63.08%). High school students were 29.23%, while infants/toddlers were 20%. The lowest percentage was 6.15% adults.

Q5 Funding Source (65 answered and 5 skipped it): Funding sources were primarily insurance funded (76.92%), followed by state funding (30.77%). Private pay was 18.46% and 9.23% marked other.

Q6 Treatment location (65 answered and 5 skipped it) was primarily at the client’s home (64.62%), followed by clinic’s setting (60%), private school (55.38%), public school (21.54%) and other 7.69%.

Q7 In what areas of treatment do you feel most competent? (59 answered and 11 skipped it) The area of treatment they felt were most competent broke down as follows, from highest to lowest percentage: treatment plan (69.49%); supervision (66.10%); behavior plans (59.32%); initial assessment (44.07%); parent training (38.98%); intake process (23.73%); and then all these above are declared to be enjoyed by (22.03%).

Q8 In what areas of treatment do you feel you can use more training? (59 answered and 11 skipped it) The area of treatment where they felt they could use more training was parent training (49.15%); followed by initial assessment (33.90%); intake process (28.81%); behavior plans (18.64%); treatment plan (16.95%); supervision (11.86%) and finally in all the above (6.78%).

Q9 Do you feel equipped in knowing what skill deficits to target in your ABA treatment? (59 answered and 11 skipped it) Form highest to lowest percentage, they felt equipped to target skill deficit 55.93% of the time; usually 16.95%; often 15.25%; always 8.47% and 3.39% would appreciate more training.

Q10 Do you feel your practice would benefit from research on the nervous system in Autism (neuroscience research) to enhance treatment planning and programs? (58 answered and 12 skipped it) 87.3% said yes and 12.07 said no.

**FIGURE 3:**
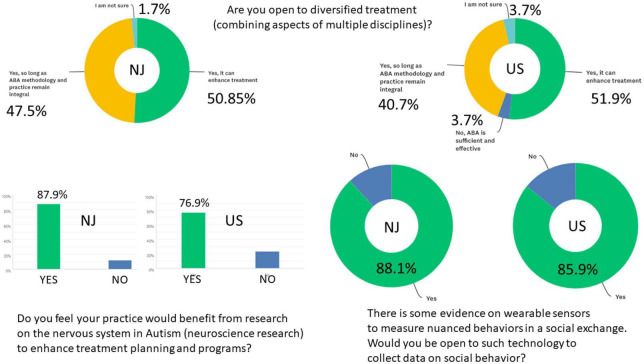
Summary of key results from the BCBAs responses in New Jersey and across the USA, on openness to diversify treatments, infusion of neuroscience research to their practices and use of wearable biosensors to measure nuanced behaviors.

Figure 3 Q11–Q13, Q11 Are you open to diversified treatment (combining aspects of multiple disciplines)? (59 answered and 11 skipped it) Here 50.85% said yes, it can enhance treatment; 47.46% said yes, so long as ABA methodology and practice remain integral; 1.69% was not sure, but none said no.

**FIGURE 4:**
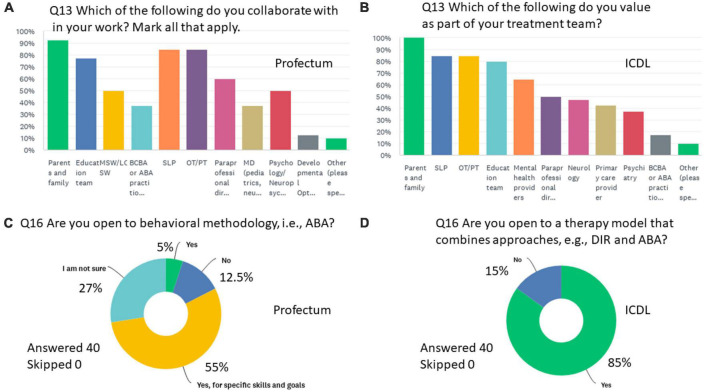
Summary of key results from the DM practitioners responding to Q13 **(A)** Form the Profectum Foundation group and **(B)** From the ICDL group. **(C,D)** Are the responses to Q16 by each of the groups, respectively.

Q12 There is some evidence on wearable sensors to measure nuanced behaviors in a social exchange. Would you be open to such technology to collect data on social behavior? (59 answered and 11 skipped it) 88.14% said yes and 11.86% said no.

To increase clarity and in the interest of space, questions Q13-Q19 are presented as topic word clouds extracted from the text using Latent Dirichlet Allocation (LDA) topic model to discover latent themes in the data and infer the word probabilities in topics. Full verbatim responses are tabulated in the Supplementary material for each of the questions Q13–19.

Q13 How would you describe differences in your treatment for young (0–5) versus older clients? (Answered: 50 Skipped: 20).

**FIGURE 5:**
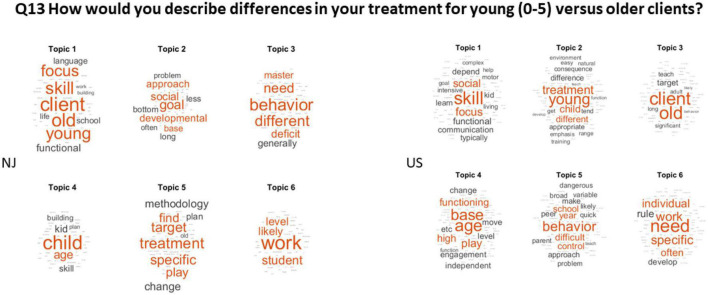
Differentiating younger from older clients consistently across NJ and the rest of the USA. The LDA revealed 6 topics in each of the data sets from NJ and the USA.

Figure 5 shows the topics with word probabilities (higher probabilities are represented by larger words in red, while low probabilities are smaller in gray) summarizing the responses in NJ and across the USA. Both groups agreed that it is easier to teach younger children (0–5) than older clients, with suggestions of more personalized approaches needed as the individual ages. They reported in both groups that younger children can master goals faster than older clients, with a focus on language skills in younger children and a focus on activities of daily living, preparing to transition and functional communication skills in older clients. Both coincide with the difficulties that challenging behaviors pose for older clients. Both groups mentioned the use of play in younger children with an integrative approach that brings to ABA some elements of the DIR^®^ Floortime methods. In adults, the focus is more functional, and some clinicians reported focusing on nuanced behaviors that could help them socially.

Q14 What additional supports do you think your client families can benefit from?

(Answered: 50 Skipped: 20).

**FIGURE 6:**
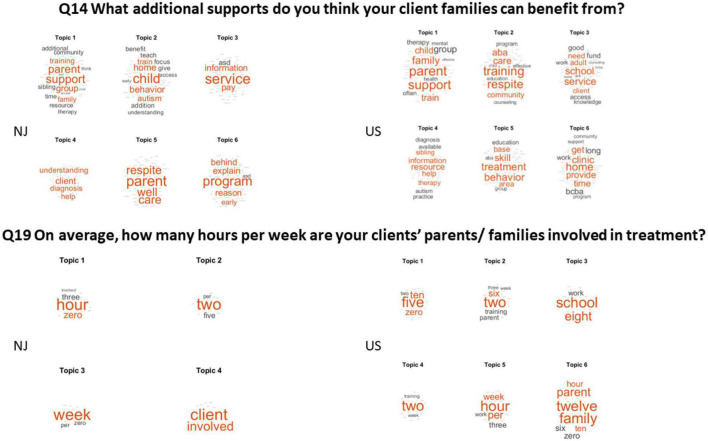
The need for parents’ support and the average number of hours that the client’s families/parents partake in treatment.

Figure 6 shows the results of question 14 on the top panel for NJ and the USA. Both groups coincided in the need of support with parents’ training and parents’ respite, parents’ mental health and support groups. Since both groups mentioned the need for more training for the families, question 19 is included in this figure as well. Both in NJ and across the USA, the client’s parents/family are involved an average of 1–2 h per week of treatment. Across the USA, some reported 1–5 and up to 8 h. However, the consensus according to the frequency of average hours reported was 1–2 h of treatment. We note that in this case, we translated the number to text, by replacing the number 1 with the word “one,” the number 2 with the word “two,” etc., and we left the dash in between (e.g., 1–2 is “one-two”) so the frequency count was consistent. Verbatim responses in numbers are in the Supplementary material.

Q15 What aspects of the job do you find most stressful?

(Answered: 53 Skipped: 17).

The most stressful part of the job in New Jersey was by far the parents’ involvement in the therapy, managing the parents’ expectations and slow progression, combined with the lack of training to follow up with the therapy off hours. This is followed by the issues with insurance coverage, whereby the practitioner needs to juggle the treatment planning time and the supervising of the therapy. This combined with the lack of support staff to help with billing and the limited training of paraprofessionals, makes for a stressful job.

Q16 What aspects of the job do you find most rewarding?

(Answered: 54 Skipped: 16).

**FIGURE 7:**
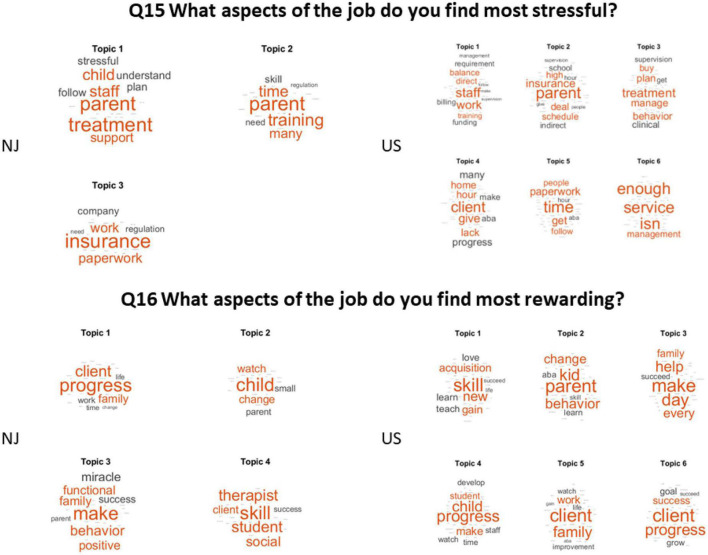
The most stressful **(top)** and the most rewarding **(bottom)** aspects of the job.

Both the New Jersey and the USA cohorts see palpable progress as the most rewarding aspect of their job, particularly with regards to children. This is reflected in the bottom panel of Figure 7.

Q17 What do you see as the greatest strength of ABA as it relates to autism treatment?

(Answered: 52 Skipped: 18).

The greatest strength of ABA was by far skills, followed by behaviors treatment and teaching (NJ), consistency and effective treatment across the USA. They also consider valuable the measurable procedures that lend consistency to the treatments and the systematic results that enable them to show evidence-based progress. BCBAs in both groups value the ability to break simple tasks in smaller parts to teach systematic patterns and being able to measure the outcomes of precise goals, with progress or regression being available through graphs.

Q18 In what areas do you see room for diversified treatment?

(Answered: 45 Skipped: 25).

**FIGURE 8:**
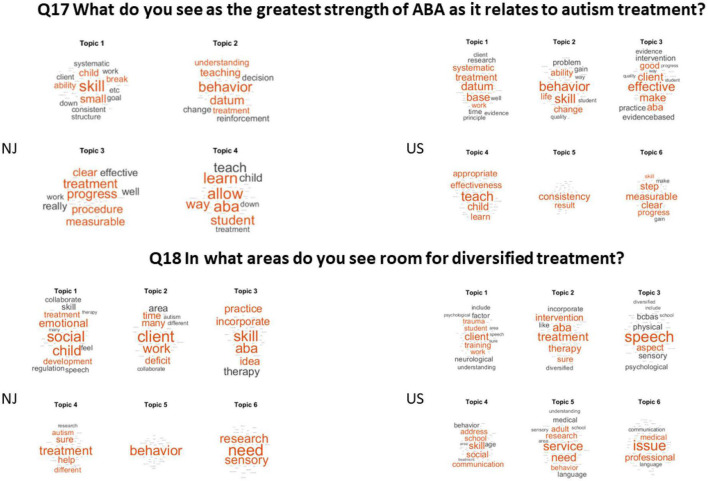
The greatest strength of ABA and the areas where there is room for diversification of treatment.

The NJ cohort sees social and emotional behaviors of the child as the area needing more diversification. Across the US, practitioners declared speech as the area needing more diversification, followed by other services and issues with treatments. In both groups there is mentioning of need to diversify treatment to address sensory issues, stereotypies and client’s general indifference (if not distaste) for social interaction” and emotional regulation, social and emotional integration, anxiety linked to comorbidities or co-existing disorders. These are shown across topics in Figure 8.

Q19 On average, how many hours per week are your clients’ parents/families involved in treatment (Answered: 53 Skipped: 17).

Here in Figure 6, we see that 1–2 h per week on average was the common most frequent answer in both the NJ and the USA cohort.

### 3.2. BCBAs’ USA survey

Q1 Do you consent to participate in this survey? (Answered: 98 Skipped: 4).

98.98% said Yes and 1.02% said No.

Q2 Zip Code: (Answered: 96 Skipped: 6) See Figure Supplementary Appendix B.

Q3 Level of ABA Certification: (Answered: 98 Skipped: 4).

RBT (1.02%); BCaBA (96.94%); BCBA (2.04%); BCBA-D (0%).

Q4 Age of treatment population: (Answered: 97 Skipped: 5).

From higher to lower percentages, school age was the largest (82.47%); pre-school (74.23%); high school (55.67%); adults (31.96%).

Q5 Funding Source: (Answered: 95 Skipped: 7).

From higher to lower percentages, insurance funding (61.05%); state funding (46.32%); private pay (38.95%) and other (17.89%).

Q6 Treatment Location (Answered: 97 Skipped: 5).

From higher to lower, the client’s home (64.95%); clinic setting (55.67%); public school (41.24%); private school (28.87%) and other (11.34%).

Q7 In what areas of treatment do you feel most competent? (Answered: 78 Skipped: 24).

From higher to lower, treatment plan (71.79%); behavior plans (66.67%); supervision (58.97%); initial assessment (52.56%); parent training (44.87%); intake process (38.46%) and all above choices (43.62%).

Q8 In what areas of treatment do you feel you can use more training?

(Answered: 76 Skipped: 26).

From higher to lower, parent training (50%); supervision (35.53%); intake process (31.58%); initial assessment (25%); all above choices (11.84%) and treatment plan (5.26%).

Q9 Do you feel equipped in knowing what skill deficits to target in your ABA treatment? (Answered: 81 Skipped: 21).

From higher to lower, most of the time (64.29%); always (14.81%); usually (11.11%); I would appreciate more training (6.17%) and often (3.70%).

Q10 Do you feel your practice would benefit from research on the nervous system in Autism (neuroscience research) to enhance treatment planning and programs? (Answered: 78 Skipped: 24).

76.92% said Yes, 23.08% said No.

Q11 Are you open to diversified treatment (combining aspects of multiple disciplines)? (Answered: 81 Skipped: 21).

5.85% said yes, it can enhance treatment and 40.74% said yes, so long as ABA methodology and practice remain integral. 3.7% said no, ABA is sufficient and effective and 3.7% said they were not sure.

Q12 There is some evidence on wearable sensors to measure nuanced behaviors in a social exchange. Would you be open to such technology to collect data on social behavior? (Answered: 78 Skipped: 24).

85.90% said yes and 14.10% said no. See Figure 4.

Q13 How would you describe differences in your treatment for young (0–5).

Versus older clients? (Answered: 73 Skipped: 29) See Figure 5.

Q14 What additional supports do you think your client families can benefit from? (Answered: 73 Skipped: 29) See Figure 5 (top panel).

Q15 What aspects of the job do you find most stressful?

(Answered: 78 Skipped: 24) See Figure 7 (top panel).

Q16 What aspects of the job do you find most rewarding?

(Answered: 78 Skipped: 24) See Figure 7 (bottom panel).

Q17 What do you see as the greatest strength of ABA as it relates to autism treatment?

(Answered: 77 Skipped: 25) See Figure 8 (top panel).

Q18 In what areas do you see room for diversified treatment?

(Answered: 73 Skipped: 29) See Figure 7 (bottom panel).

Q19 On average, how many hours per week are your clients’ parents/families involved in treatment?

(Answered: 74 Skipped: 28) See Figure 6 (bottom panel).

### 3.3. Developmental model responses

Q8 In what areas of treatment do you feel most competent?

They listed relationships as the area of least strength or most difficulty to teach. Yet, supporting relationships was listed as an area of great satisfaction. Moreover, self-reporting competence in research areas ranked highest in the areas of relationships. Lastly, parental stress, linked to relationships, was listed frequently as an area of greatest stress. The percentage breakdown was as follows:

• Knowledge of Development 43.53% felt strongest in this area.
• Knowledge of individual differences 41.18% felt strongest in this area.
• Knowledge of relationships 15.29% felt strongest in this area.

Q9 Identify strategies/techniques for working with children within the DIR model.

The listed areas such as identifying and targeting deficits, working/playing with the child, and parent coaching are all included as developmental tools in the DIR^®^ program. Behavior management was a common complaint, also listed as strategies, yet this is not generally included as part of DIR philosophy and training. Their philosophy believes that through adequate use of strategies and good intervention at every developmental level, there will be little need for behavior management. This may explain their higher client groups among toddlers 68.82%, pre-school 87.65% and young children of school age (78.24%), whereas adults make up only 16.47%.

59.41% listed working with the child and strategies for play as their most comfortable skill.

22.94% listed identifying and targeting specific deficits as their most comfortable skill.

13.53% listed parent coaching as their most comfortable skill. According to DIR training, knowledge of relationships plays closely into parent training and coaching.

3.53% listed behavior management as their most comfortable skill.

Q10 Do you value interdisciplinary collaboration? Which of the following do you collaborate with in your work? Mark all that applies.

DIR practitioners report to highly value interdisciplinary collaboration. They mentioned highly interdisciplinary treatment teams. Notably, interdisciplinary value diminishes to less than 50% of respondents when asked about paraprofessionals (the Registered Behavioral Technicians, RTBs). The RBTs spend the bulk of time during school hours interacting with the autistic individuals. They do not value as high the primary care providers, psychiatry, and neurology. Interesting, they value BCBA input, with 21.76% stating that the BCBA input was valuable. The percentage breakdown is as follows:

95.88% valued parents and family as part of team approach.

87.06% valued OT/PT input.

81.76% valued SLP input.

74.71% valued the education team.

64.12% valued mental health providers.

49.91% valued primary care providers.

48.24% valued paraprofessionals.

41.18% valued psychiatry.

40.59% valued neurology.

21.76% valued BCBA.

15.29% specified other areas of valuable input (audiologists, music therapists, crania sacral practitioners and others).

Q11 Are you informed on advances in Neuroscience research?

Several research areas were listed, and respondents chose to measure as “very informed” vs. “somewhat informed.” No objectivity in these answers could be assessed as they are self-reports on self-perception by the respondents.

In the area of relationships, 44.71% listed themselves as very informed; 30% listed themselves as somewhat informed.

In the area of Neuroscience, 42.94% listed themselves as very informed (73 respondents); 35.29% listed themselves as somewhat informed.

In the areas of trauma and stress, 34.91% list themselves as very informed; 40.83% list themselves as somewhat informed.

In the area of Medical Research, 29.41% list themselves as very informed; 55.88% list themselves as somewhat informed.

In the area of Behavioral Research, 40.83% list themselves as very informed; 43.79% considered themselves somewhat informed. We note that a combined total of ∼84% considered themselves at least somewhat informed in this area, an area of knowledge that nevertheless only 21% value as part of their treatment team.

Q12 Do you feel supported by your work settings?

69.41% of respondents worked in settings which support developmental approaches. 22.94% felt somewhat supported in their work setting.

4.71% did not really feel supported.

2.94% did not feel supported in their work settings.

Q13 Are you open to a therapy model that combined approaches, e.g., DIR and ABA?

82.74% of respondents were open to a model which combines approaches.

17.26% were not open to this.

Q14 There is some evidence on wearable sensors to measure nuanced behaviors in a social exchange. Would you be open to such technology to collect data on social behavior?

85.29% were interested in sensors to measure stress and connectedness during an interaction.

14.71% were not interested in this option.

Q15 What age range do you work with? (Out of 170 participants).

32.35%, 55 respondents specified 0–3.

37.6%, 64 respondents specified 2–5.

17.05%, 29 respondents specified 3–6.

3.5%, 6 responded 5–7.

1.17%, 2 responded 6–9.

0.58%, 1 responded13–15.

1.17%, 2 responded 18–21.

**FIGURE 9:**
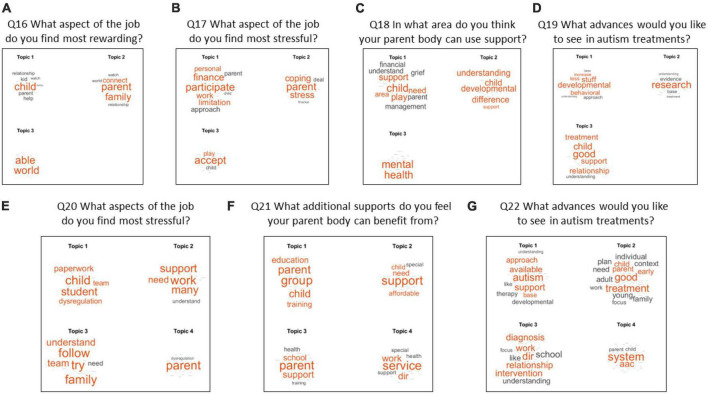
Themes revealed from questions 16–22 show 3–4 topics from the DM groups combined. Panels **(A–G)** cover questions 16–22.

Questions 16–22 are summarized in topics uncovering probability word maps in Figure 9.

Q16 What part of your work do you find most rewarding?

Most frequent theme was building relationships and supporting connection, making progress, and offering hope and help to families.

Q17 What part of your work do you find most stressful?

Several main themes emerged here: Families accepting the diagnosis; Financial stress; participating in therapies with different philosophies and coping with family, or autism-related difficulties.

Acceptance of the diagnosis and participation in treatment (31 responses).

Coping with family, disabilities, or family stress (34 responses).

Difficulties surrounding conflict between approaches in autism; resulting in a lack of comprehensive approach (49 responses).

Difficulties related to the limitations of practice or service ability- inability to meet the need, inability to address specific issues (43 responses).

Personal work stress- paperwork, administration issues, time limitations etc. (54 responses).

Difficulty with judgment of outsiders toward the disability (2 responses).

Q18 Name some elements that interfere with therapy.

These responses were like those causing stress. Additional components included concerns about the parents’ mental health, understanding the DIR^®^ approach, and families struggling with rate of progress.

Q19 How could your work improve?

14 responses fell within the area of improving family support.

14 responses called for more combined approaches and interdisciplinary work, but without specifying which discipline.

17 responses called for a reduction in behaviorally based work on ABA.

47 responses called for an expansion of relationship or developmentally based work. Relationships were often specified instead of development.

39 referenced furthering research. The topics to be researched varied from increased DIR^®^ research to greater SI research, to more neuroscience research, and several other areas mentioned.

66 responses were hard to classify and were not tagged as part of a category.

Q20 Are you open to behavioral methodology, i.e., ABA?

60% Yes, for specific skills and goals.

27% I am not sure.

10% No.

3% Yes.

## 4. Discussion

In this work, we set to survey two complementary approaches to ASD, boasting opposing views on how to treat the condition. We enlisted people in each field to design and launch surveys using Survey Monkey software, upon approval of their certification/accreditation boards. Two sets of questions were distributed, one for applied behavioral analysis (ABA) designed by Board Certified Behavioral Analysts (BCBAs) and the other by developmental model (DM) clinicians (trained in DIR^®^ Floortime.) The former attracted 70 BCBAs from New Jersey and 102 from the USA, while the latter attracted 6 DM from New Jersey and 168 from the USA and Canada. The surveys were slightly different, yet both made inquiries about the possibility of diversifying treatments, infusing new Neuroscience content in their curricula and using wearable biosensors to capture nuance aspects of behaviors and relationships. They also commented on the most rewarding and the most stressful areas of their practices, and their overall desire to collaborate with each other.

Their answers to these surveys revealed that most practitioners feel that the time is ripe for collaborative work and treatment diversification. Most participants in both groups would also readily welcome new scientific and technological advances to their practices. Importantly, most find joy in seeing progress and improvements in their clients but acknowledge several stressful areas that need to be recognized by their institutions and by society in general. Most notably, was the amount of paperwork they need to complete to attain insurance coverage, with limited staff support and working under pressure of having to word the activities and codes correctly to not be fraudulent in their practices. This portion of the responses revealed a conscientious army of fellows willing to do the right thing in both groups.

Both groups feel that parents of autistic people across all ages need more respite, more support for their own mental health challenges and more training to support the therapies after the therapists finish with their home-therapy hours. Indeed, across the USA, an average of only 1–2 h per week are shared with the parents, while both interventions require complex social and emotional regulation of the child and care givers. Although the BCBAs seem to cover a broader range of ages than the DM therapists, both agree that younger clients make faster, more palpable progress than older clients, who need more personalization of the intervention. This once again confirms the importance of starting early, though reliance on a one-size-fits-all model in early childhood can be dangerous and even lead the nascent nervous system astray.

The young system progresses at an accelerated, non-linear rate, yet under observational means, no therapy can currently decouple changes that are caused by the therapy itself, from changes that are due to natural progression of early neurodevelopment. At the start of life, the system is highly plastic, yet some interventions may not properly leverage this plasticity or even fail to reveal important latent changes occurring largely beneath the therapist’s awareness, away from the naked eye’s detection. Whether the perturbation/intervention might (implicitly) cause harm or not is a different question that current subjective manualized methods are ill-equipped to address. High sampling resolution sensors compatible with the refreshing rates of the sensory and motor systems will be necessary to address the important question of decoupling gains from the therapy and gains from natural progress of a coping nervous system, evolving along different and unexpected paths.

### 4.1. A word on biosensors

There is a plethora of biosensors today that can help track several physiological signals and functions of the nervous systems. Among them some have received FDA approval and others are still under testing/developing phase, gathering data form the population to discover various optimal parameters and their ranges to track progression of a disorder and/or therapeutic outcomes. Some of the biosensors provide information about heart rate variability amenable to track states of the heart during the day e.g., using methods in Figure 2E revealing flight-or-fight states (Elsayed and Torres, 2023) or in other cases catatonic states (as when there are excessively high signals in the high-frequency bands). Other biosensors measure motion (Torres et al., 2013a) and can identify excessive noise and randomness in the motor code as that shown in Figures 2A–C. Others quantify prolonged latencies in sound processing even at neonatal stages (Torres et al., 2023), thus impacting sensory processing and integration whereby despite attempts of the child to control behavioral states, we can see the excess involuntary randomness.

Other sensors can capture levels of tremor (Torres and Denisova, 2016) and changes in the involuntary motor signatures across the lifespan (Torres et al., 2020a). Furthermore, using Apps in combination with smartphones and smart shoes, it is possible to predict issues with gait, balance, and vestibular problems (Torres et al., 2018; Bermperidis et al., 2021). Other video-based estimation models can also provide various (non-invasive) data acquisition scalable means to inform about eye gaze, facial micro expressions, universal muscle units of the face and help infer various emotional or attentional states of the person alone, and of the interacting dyad (in the case of therapist and child) (Bokadia et al., 2020). These biometrics are important during therapies to track for example, the amount of rapport and synchronicity of the participants, the predispositions of the child to follow instructions, or the exploratory states of the child to help the child learn more effectively, etc.

In terms of their accuracy, some sensors have been tested broadly and can make accurate predictions. For example, the Apple watch can now predict signatures of Parkinson’s disease^5^ and various Apps discussed within the movement disorders society can help patients track their daily routines, sleep, seizures, medication intake, mood, among others, e.g., see https://www.strive.group/. Among FDA approved sensors are pulse oximeters (Browne et al., 2021) leveraging photoplethysmography in smart phones or watches like the Apple watch. Others can be used for cardiorespiratory assessments (Pinsky et al., 2016).

Reviewing this literature is beyond the scope of this paper. However, biosensors are routinely used in other fields of research and medical practice. Unfortunately, in autism, this has not been the case, although these surveys reveal some awareness by therapists and willingness to try out some of these new emerging technologies.

Despite shared awareness about biosensors and the need for updating their curricula, the NJ and the USA BCBAs responded slightly differently to the question of diversification of treatment. While those from NJ seek diversification of treatments to enhance social and emotional behaviors, those from across the USA seek diversification toward treatments that enhance speech issues. Unbeknown to both groups are the foundational basic sensory-motor units scaffolding both social-emotional learning and the development of speech/communication, because their curricula do not offer content on these developmental aspects of the nervous system. Indeed, not even basic courses on neuroanatomy and neurophysiology teaching them about structure and function of the human nervous system’ development exist in their current curriculum. This is also the case in the DM curricula, yet they offer this knowledge through webinars and continuing education courses that are also taken by BCBAs on their own. The latter are insufficient to provide clinicians with a solid scientific and technical foundation that prepares them adequately to understand the complex evolution of the nervous system toward social, emotional, and communication abilities. In this sense, if their accrediting bodies and leaderships do not come up with actionable steps, both communities will be missing a terrific opportunity for transformative change bringing them up to speed with the new demands that the shifts in diagnosis have brought to the ecosystem of autism.

### 4.2. Consequences of a monolithic approach to ASD

The broadening of ASD diagnosis criteria paired with lobbying forces advocating exclusively for ABA over any other therapy resulted in a very profitable monopoly that generated what has come to be known as the Autism Industrial Complex (Broderick, 2022): A multi-billion-dollar industry that cannot possibly absorb the needs of the population. With 1/36 children of school age on the spectrum,^6^ under state laws that mandate insurance coverage in most cases and do so under a decentralized service provider system, the profits have been so excessive that Private Equity (PE) firms have recently come into the picture of autism and started to dismantle its ABA conglomerates across the US.

Under the PE aggressive growth tactics, families suffer because practitioners become underpaid and overworked; insurers cut reimbursement and the quality of the therapy dilutes. In a system that is already stressed (as revealed by these surveys), with understaffed centers and therapies working under intense pressure to succeed, such interference will become a failed model across the USA.

Cutting costs to increase profitable margins, PE firms will drive ABA practices further into trouble and spoil their reputation in several sectors of the population. While all fifty states in the United States have adopted insurance mandates for coverage of services for autism, the shortage in qualified providers exacerbates the problem nationwide. Most of these mandates include or require ABA services, which has boosted the market for ABA and allowed it to be seen as a default standard of care narrowly defined for what is today a wide spectrum of conditions. A recent article further explaining these issues, “*As Private Equity Comes to Dominate Autism Services,”* by historian John Summers (see Supplementary Appendix Notes 6, 7), explains that, as a representative example of this nationwide ABA phenomenon, the insurance mandate in Massachusetts has set no limit in terms of age and amount of money for billing. In his state, as further explained in a podcast episode (by co-author Hannah Varkey, see Supplementary Appendix Note 9), PE firms are attracted by this lucrative ABA market in instances like Blackstone acquiring the Center for Autism and Related Disorders and Rothschild obtaining New England ABA.

An area that has been critical of ABA is the military insurer TRICARE, which produced a report about fiscal year 2019 whereby the Department of Defense alone spent $1.53 Billion on ABA services to almost 16,000 individuals (averaging $23,253 cost per participant.) However, in this, the largest up to date study of ABA, with 3,794 participants, it was concluded that ABA services are not working. Adding the PE intrusion will further compound the problem and reduce resources for continuing education and proper training of the practitioners.

Without proper training, practitioners will miss early markers of developmental derailment, because many milestones in early stages of human neurodevelopment escape the naked eye of an untrained observer. Even the trained clinicians will miss nuance information embedded in the continuous streams of biorhythmic activities generated by the nervous system which is occurring at a micro-level of behavior escaping the naked eye. Such limitations can be mitigated by modern instrumentation that is commercially available today thanks to two contemporary developments: the wearable biosensors and the neuroscience revolutions.

A possible solution to diversification of treatments and access to treatments at scale might be to expand legislation to cover other forms of treatment in autism, under a more centralized format that sets up standards based on the latest scientific and technological advances of medicine and physiology. This approach would enable repurposing for autism existing solutions in other fields, while increasing surveillance by other, more technical fields of Science Technology Engineering and Math, where objective quantification of treatment outcomes already exists and a more rigorous treatment of science is in place, one that allows for openness, transparency, and reproducibility of our outcomes.

### 4.3. Moving forward together

How can we use the information generated by these surveys to improve services and treatments by these clinicians to autistic children and their family?

An important area to address the challenges and interests expressed by both surveys would be to educate the clinical professionals. One possible avenue would be to enhance curricula for certified professionals in both ABA and developmental practices. For ABA professionals, added areas of focus could include basic neuroanatomy and neurophysiology, general principles of neuroscience and in particular, principles of developmental neuroscience. Moreover, it is possible to leverage conventional knowledge already offered at Psychology programs by adding it to ABA curricula. These would include classes on Sensation and Perception (Wolfe et al., 2020), and training techniques assessing relationship-based practices in young infants. Other techniques translating recent findings in exploratory vs. error correction learning [e.g., (Vaskevich and Torres, 2022)] will be useful, but above all, highly multi-disciplinary curricula should include early markers of neural intactness that will later influence language development, sensory processing and motor control as basic foundational blocks of social exchange and communication (Chambers et al., 2020; Seif et al., 2021; Hines et al., 2022; Torres et al., 2023).

In addition to the above, curricular activities for developmental practitioners should also include systematizing the quantification of behavior, learning to build hierarchical structures of aspects of behavior that are conducive of instruction readiness, emotional self-regulation, and self-control of attentional windows, among other aspects of behaviors that the young infant will need to mature by the time that attends school and receives instructions.

Both groups will benefit from incorporating in their curricular activities a practicum involving the use of e.g., FDA-approved wearable sensors to collect “benchmark” data. Longitudinal studies of such data can then be used to separate therapeutic gains from natural physiological gains that neurodevelopment produce -a common confound in all current therapies.

As both fields now rely on paper-and pencil-data and on reports to quantify progress and treatment efficacy, most nuanced aspects of behaviors are lost, because they escape the naked eye of the observer. Human error and subjective opinion can confound the process as well, since paper trail of progress is dependent on individual interpretation and does not include the child’s neurophysiological and neuroanatomical progression. Wearable sensors or other biophysical markers could complement the human subjective data and provide a more complete scientific measure of the child’s developmental progression.

Lastly, policies from insurance providers and other entities (such as lawmakers) can shift the climate toward a quality-based model of intervention supporting the clients and families. For example, limiting the maximum number of clients per BCBA based on number of work hours per week; mandating that a percent of company profits go toward quality assurance and above-mentioned training of providers; and supporting families with respite, mental health, and other services. By working together from all ends of the service spectrum we can improve the quality of care and thereby support the life potential of families and individuals with autism spectrum disorders.

## 5. Conclusion

This work reveals that in autism, the main competing therapies are ready for change that updates their practices to cope with the new influx of diagnosed infants, toddlers, and children with sensory and motor issues. Two decades ago, clinicians and researchers in the field did the best they could with what they had, a set of techniques grounded in behavioral advances dating back to the decades spanning from the 1950s–1970s. Today, new discoveries accelerated by the decade of the brain and the brain initiatives, paired with the technological advances in communication, memory storage, battery-life, and overall miniaturization of biosensors, and by accompanying computational, algorithmic advances, make the time ripe for positive change. These folks are willing and eager to move their field forward through collaborative exchanges. Hats off to their efforts, organization, and love for the job. It is up to their leadership and accrediting bodies to enable their agency to push forward this incredibly important and necessary Autism Renaissance.

## Data availability statement

The datasets presented in this study can be found in online repositories. The names of the repository/repositories and accession number(s) can be found below: https://zenodo.org/record/7968707 and https://zenodo.org/record/8125953.

## Ethics statement

The studies involving human participants were reviewed and approved by the Rutgers University Institutional Review Board. The participants provided their written informed consent to participate in this study.

## Author contributions

ET, GT, and MK contributed to the conception and design of the study. HV, RR, ME, and JT organized the database. ET performed the statistical analysis, plots, and figures. ME performed the heart rate variability analyses and scalogram plot. HV did the analyses of the demographics by geographical region and made the map plots. ET wrote the first draft of the manuscript. GT, MK, RR, ME, and HV edited the sections of the manuscript. All authors contributed to manuscript revision, read, and approved the submitted version.

## Footnotes

1 With the exception of the state of NJ, which now covers DIR/Floortime services through Medicaid plans funded by the Federal and State governement.

2
https://www.ncsl.org/health/autism-and-insurance-coverage-state-laws

3 https://www.youtube.com/watch?v=t0QsFhFpPSE and https://www.youtube.com/watch?v=8VkMgR1pv1Q

4
https://eagletonpoll.rutgers.edu/press-release-program-topic/autism/

5
https://www.smartnews.com/p/4567666424218126592?placement~=~article-preview-social&utm_campaign~=~sn_lid%3A4567666424218126592%7Csn_channel%3Acr_en_us_top&utm_source~=~share_ios_email&share_id~=~4ZzaRr

6
https://www.cdc.gov/media/releases/2023/p0323-autism.html
