# Supplementary material for: The time is ripe for the renaissance of autism treatments: evidence from clinical practitioners
Source: Front Integr Neurosci. 2023 Aug 4;17:1229110. doi: 10.3389/fnint.2023.1229110 (PMC10437220; doi:10.3389/fnint.2023.1229110)
Supplement: Supplementary file 1 [file Data_Sheet_1.pdf]

The Time is Ripe for the Renaissance of Autism Treatments: Evidence from Clinical Practitioners

Elizabeth B Torres [1,2,3*], Goldie Twerski [4], Hannah Varkey [1,] Richa Rai [1], Mona Elsayed [1], Miriam Katz [5], Jillian Tarlowe [1]

[1]Sensory Motor Integration Laboratory, Department of Psychology, Rutgers the State University of New Jersey, City Piscataway, NJ, USA

[2]Rutgers Center for Cognitive Science, Rutgers the State University of New Jersey, City Piscataway, NJ, USA

[3]Rutgers Center for Biomedicine Imaging and Modeling, Department of Computer Science, Rutgers the State University of New Jersey, City Piscataway, NJ, USA

[4] The Monarch Center, Lakewood, NJ, USA

[5] MTK Therapy, Yahalom NJ, Family Advocacy and Support, Agudas Yisroel of America, Lakewood, NJ, USA

***Correspondence:**
Corresponding Author
ebtorres@psych.rutgers.edu

Keywords: autism, applied behavioral analysis, DIR Floortime, developmental model, neurodevelopment, wearable biosensors

1 Supplementary Materials

1.1 The following questions designed by the BCBAs were asked in both the NJ and the nationwide survey:

Q1 Do you consent to participate in this survey?

Q2 Zip code

Q3 Level of ABA Certification

Q4 Age of treatment population

Q5 Funding Source

Q6 Treatment location

Q7 In what areas of treatment do you feel most competent?

Q8 In what areas of treatment do you feel you can use more training?

Q9 Do you feel equipped in knowing what skill deficits to target in your ABA treatment?

Q10 Do you feel your practice would benefit from research on the nervous system in Autism (neuroscience research) to enhance treatment planning and programs?

Q11 Are you open to diversified treatment (combining aspects of multiple disciplines)?

Q12 There is some evidence on wearable sensors to measure nuanced behaviors in a social exchange. Would you be open to such technology to collect data on social behavior?

Q13 How would you describe differences in your treatment for young (0-5) versus older clients?

Q14 What additional supports do you think your client families can benefit from?

Q15 What aspects of the job do you find most stressful?

Q16 What aspects of the job do you find most rewarding?

Q17 What do you see as the greatest strength of ABA as it relates to autism treatment?

Q18 In what areas do you see room for diversified treatment?

Q19 On average, how many hours per week are your clients' parents/ families involved in treatment?

1.2 The following question were asked by the DM clinicians:

Q1 Do you consent to participate in this survey?

Q2 Zip Code

Q3 Backgrounds of the participants

Q4 Graduate level

Q5 Levels of DM training

Q6 Age of treatment population

Q7 Treatment location

Q8 In what areas of treatment do you feel most competent?

Q9 Identify strategies/techniques for working with children within the DIR model.

Q10 Do you value interdisciplinary collaboration?

Q11 Are you informed on advances in Neuroscience research?

Q12 Do you feel supported by your work settings?

Q13 Are you open to a therapy model that combined approaches, *e.g.,* DIR and ABA?

Q14 There is some evidence on wearable sensors to measure nuanced behaviors in a social exchange. Would you be open to such technology to collect data on social behavior?

Q15 What age range do you work with?

Q16 What part of your work do you find most rewarding?

Q17 What part of your work do you find most stressful?

Q18 Name some elements that interfere with therapy.

Q19 How could your work improve?

1.3 Zip Code Geographic NJ Locations of Respondents to the Surveys.

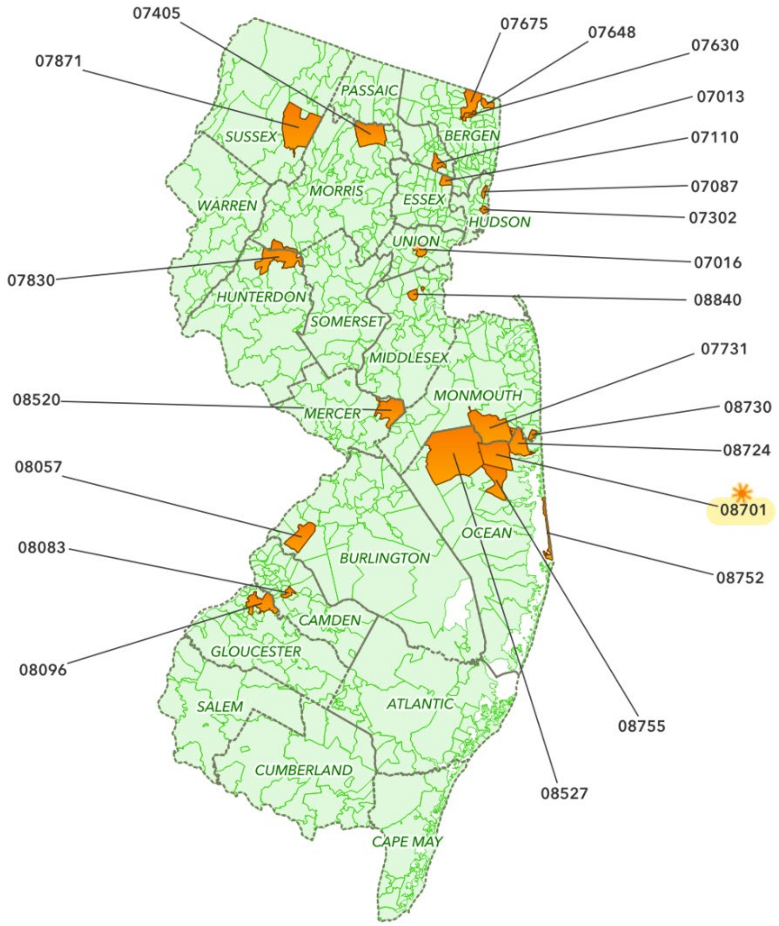

Appendix Figure 1: Map of the state of New Jersey, indicating the zip code tabulation areas of the survey respondents. Out of 69 responses, highlighted zip code 08701 (Lakewood, NJ) had the most number of responses at 39. The responses appear well distributed across New Jersey, coming from counties of various regions— Northeastern (Bergen, Morris, Essex, Passaic, Hudson, Union), Northwestern (Sussex), Central (Hunterdon, Middlesex, Mercer), Coastal (Monmouth, Ocean), and Southern (Burlington, Camden, Gloucester).

1.4 Zip Code Geographic US-Canada Locations of Respondents to the Surveys.

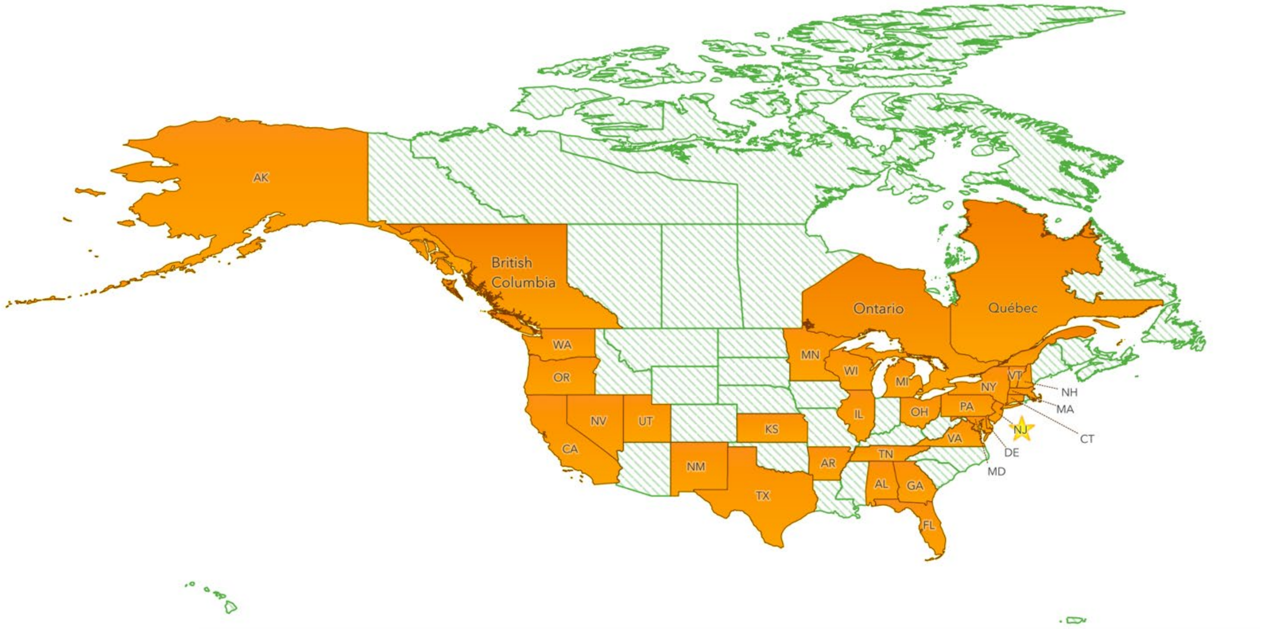

Appendix Figure 2: Map of Canada and the United States, outlining their provinces and states respectively.
The survey responses came from the 32 regions colored in orange. One response was received from French Polynesia, which is not shown here. The total survey responses received were 159, where 3 responses had unknown/ unavailable zip codes and were not included in the maps and tables.

1.5 Appendix Table 1: Survey Respondents from New Jersey

Zip Code	New Jersey Cities	No. of survey respondents
08083	Somerdale, NJ, Hi-Nella, NJ, Gloucester Township, NJ	1
07013	Clifton, NJ, Woodland Park, NJ	1
08527	Jackson Township, NJ, Cassville, NJ, Whitesville, NJ	8
08701	Lakewood, NJ	39
07630	Emerson, NJ, Oradell, NJ, Haworth, NJ	1
07405	Kinnelon, NJ, Butler, NJ, West Milford, NJ, Rockaway Township, NJ	1
07302	Jersey City, NJ	1
07871	Sparta Township, NJ, Byram Township, NJ, Jefferson, NJ	1
08730	Brielle, NJ	1
07675	Westwood, NJ, River Vale, NJ, Old Tappan, NJ	1
08724	Brick Township, NJ, Wall Township, NJ	1
07110	Nutley, NJ	1
08520	East Windsor, NJ, Hightstown, NJ, Windsor, NJ, Cranbury, NJ, Etra, NJ, Robbinsville Township, NJ	1
08057	Moorestown, NJ, Mount Laurel Township, NJ, Delran, NJ	1
08752	Seaside Park, NJ, Berkeley Township, NJ	1
07830	Califon, NJ, Tewksbury, NJ, Lebanon Township, NJ, Washington Township, NJ, Fairmount, NJ	1
07731	Howell Township, NJ, Wall Township, NJ	1
07087	Union City, NJ, Jersey City, NJ, North Bergen, NJ	2
08755	Toms River, NJ	1
07016	Cranford, NJ	1
07648	Alpine, NJ	1
08840	Metuchen, NJ, Woodbridge Township, NJ, Robinvale, NJ	1
08096	Woodbury, NJ, Deptford, NJ, West Deptford, NJ	1

1.6 Table 2: Total Survey Respondents across the world

State / Province / Region, Country	Zip Code	Number of survey respondents
Alabama, United States	36867	1
Alaska, United States	99709	1
Arkansas, United States	72762	1

British Columbia, Canada	V3H 0B3	1
	V3K 6Y4	1
California, United States	93401	1
	93536	1
	91764	1
	90404	1
	95827	1
	90808	1
	90292	1
	95240	1
	95765	1
Connecticut, United States	06611	1
	06854	1
	06057	1
Delaware, United States	19702	1
Florida, United States	32303	1
	32905	1
	33065	1
	32566	1
	33433	1
	33455	1
Georgia, United States	31501	1
Illinois, United States	60659	1
	62901	1
	61614	1
Kansas, United States	66046	1
Leeward Islands, French Polynesia	98732	1
Maryland, United States	21228	1
Massachusetts, United States	01876	1
	02767	1
	02131	1
	01020	1
	01915	1
Michigan, United States	49002	1
	49442	1
	48202	1
Minnesota, United States	56303	1
Nevada, United States	89107	1

New Hampshire, United States	03077	1
New Jersey, United States	08083	1
	07013	1
	08527	8
	08701	39
	07630	1
	07405	1
	07302	1
	07871	1
	08730	1
	07675	1
	08724	1
	07110	1
	08520	1
	08057	1
	08752	1
	07830	1
	07731	1
	07087	2
	08755	1
	07016	1
	07648	1
	08840	1
	08096	1
New Mexico, United States	87120	1
New York, United States	10314	1
	11230	1
	12901	1
	10110	1
	12857	1
	10028	1
	10952	1
	10016	1
	14478	1
	12345	1
Ohio, United States	43221	1
Ontario, Canada	L4L 8C3	1
	L9N 0P5	1

	K2J 5T3	1
Oregon, United States	97214	1
Pennsylvania, United States	18201	1
	15202	1
	18707	1
	19040	1
	19533	1
Québec, Canada	H1M 3J8	1
Tennessee, United States	37043	1
	37660	1
	37204	1
Texas, United States	77406	1
	78665	1
	78227	1
	78109	1
	78245	1
Utah, United States	84414	1
	84040	1
Vermont, United States	5086	1
Virginia, United States	22902	1
	23509	1
	23111	1
Washington, United States	98103	1
	98034	1
	98056	1
	98501	1
	98404	1
	98606	1
Wisconsin, United States	54840	1
	53213	1
	54701	1

1.7 Notes (references used to build the maps):

1. Steven Manson, Jonathan Schroeder, David Van Riper, Tracy Kugler, and Steven Ruggles. IPUMS National Historical Geographic Information System: Version 17.0 [dataset]. Minneapolis, MN: IPUMS. 2022. http://doi.org/10.18128/D050.V17.0

2. QGIS.org, 2023. QGIS Geographic Information System. QGIS Association. http://www.qgis.org

3. County Boundaries of NJ, Hosted, 3424. *NJ Geographic Information Network*. map. Retrieved from https://njogis-newjersey.opendata.arcgis.com

4. Statistics Canada. (2011, November 29). Canadian Province and Territory Cartographic Boundary Shapefile - 2011 Census. *USGS ScienceBase - Catalog*. map. Retrieved from https://www.sciencebase.gov/catalog/

5. Cartographic Boundary Files - States. (2021). *United States Census Bureau*. map. Retrieved from https://www.census.gov/en.html

6. Summers, J. (2021, April 2). As Private Equity Comes to Dominate Autism Services…. The Nation. Retrieved from https://www.thenation.com/article/society/private-equity-autism-aba/.

7. Summary - Autism and Insurance Coverage State Laws. National Conference of State Legislatures. (2021, August 24). Retrieved from https://www.ncsl.org/health/autism-and-insurance-coverage-state-laws

8. Ducharme, A. (2021, June). The Autism Mandate in America State By State. Bierman Autism Centers - Founded in 2006. Retrieved from https://www.biermanautism.com/resources/blog/autism-insurance-mandate-by-state/

Varkey, H. The World of Autism: A Critical and Historical Analysis (with Dr. John Summers). Autism Thinks. episode. Retrieved from https://podcasts.apple.com/us/podcast/the-world-of-autism-a-critical-and-historical/id1513218672?i=1000580788570
